# Walnut Allergy in Children: A Diagnostic Test Accuracy Study

**DOI:** 10.1111/cea.70078

**Published:** 2025-05-14

**Authors:** Mattia Giovannini, Camilla Fazi, Martina Tesi, Giulia Liccioli, Lucrezia Sarti, Simona Barni, Leonardo Tomei, Benedetta Pessina, Claudia Valleriani, Robert Boyle, Riccardo Pertile, Francesca Mori

**Affiliations:** ^1^ Allergy Unit Meyer Children's Hospital IRCCS Florence Italy; ^2^ Department of Health Sciences University of Florence Florence Italy; ^3^ Immunology Laboratory Meyer Children's Hospital IRCCS Florence Italy; ^4^ National Heart and Lung Institute Imperial College London London UK; ^5^ Department of Clinical and Evaluative Epidemiology Trento Health Service Trento Italy

**Keywords:** anaphylaxis, children, cut‐off values, oral food challenge, prick‐by‐prick, specific IgE, walnut allergy

**Summary**

- Prick‐by‐prick test to walnut had a similar diagnostic performance to specific IgE, slightly better for specific IgE.
- The diagnostic cut‐offs identified in our study need validation in other populations.

To the Editor,

The prevalence of oral food challenge (OFC) confirmed food allergy to tree nuts is evaluated to be less than 2%, including children [1]. Tree nuts IgE‐mediated reactions have the potential of being clinically severe and are one of the leading causes of fatal anaphylaxis [2].

Walnut is one of the allergens responsible for nut allergy, diffused in Europe, especially in the Mediterranean area [3]. Jug r 1 is a major component allergen in young children with clinical walnut allergy [4].

Walnut allergy diagnosis is based on clinical history, examination and specific testing by using skin prick test, prick‐by‐prick test (PbP) and/or serum specific IgE (sIgE) and, where necessary, oral food challenges (OFC) [5]. OFC is potentially at risk for severe anaphylaxis, and it takes considerable time [6]. In this study, we evaluated the diagnostic test accuracy of PbP and sIgE to walnuts in children.

From January 2020 to September 2022, charts from patients referred to the Allergy Unit of Meyer Children's Hospital IRCCS who had a positive PbP or sIgE to walnuts were revised, as part of a nut allergy workup or because of a suspected reaction to walnut. All children who performed an OFC to walnuts were included in the study, and written informed consent was obtained from the children's parents for all procedures performed.

PbP to walnut and other nuts (peanut, hazelnut, pine nut, almond, cashew and pistachio) was performed with fresh nuts in all included patients [7]. PbP was considered positive if the wheal diameter was ≥ 3 mm at the 15 min reading. Normal saline and histamine (10 mg/mL; Alk Abellò) were used as negative and positive controls. sIgE was determined using a commercial assay (ImmunoCAP system, Thermo Fisher Scientific).

OFCs were open and not placebo‐controlled, using 5 mg of walnut (0.52 mg of walnut protein) as the starting dose; then, the dose was doubled every 20 min until a reaction occurred or the total amount of about 5 g of walnuts (about 520 mg of walnut protein) was reached according to a modified protocol [8]. As soon as any objective clinical manifestations were observed, the reaction was treated, and the OFC was stopped [5]. OFC evaluators were not blind to PbP and sIgE results. Epinephrine dispensation was used according to pertinent guidelines [9].

Eighty‐nine children satisfied the inclusion criteria, of whom 45 were walnut tolerant and 44 walnut allergic, including eight patients with anaphylaxis, of whom four received intramuscular adrenaline.

Mean age at OFC was 106.1 months (SD 44.7 months) in walnut tolerant children and 114.6 months (SD 48.8 months) in those with walnut allergy. Gender was also similar, with 71% male in the walnut tolerant group and 68% male in the walnut allergic group.

Most of the children (75%) were atopic. In the walnut tolerant group, 21/45 (46%) patients had a previous history of walnut reaction and the mean time from the reaction to OFC was 40.3 months (SD 32.8 months), whereas in the walnut allergic group, 28/44 (63%) patients had a previous history of walnut reaction and the mean time from the reaction was 61.5 months (SD 36.2 months). The difference in reaction history between allergic and tolerant patients was not statistically significant (*p* > 0.05).

All children were also examined for other nuts. In the walnut‐tolerant group, 26/45 (58%) had a history of reaction to other nuts. In the walnut‐allergic group, 19/44 (43%) had a history of reaction to other nuts.

There were divergences in PbP and sIgE to walnut between tolerant and allergic participants. PbP median diameter was 4.0 mm (IQR 3.0 mm) and median sIgE was 2.1 kUA/L (IQR 4.9 kUA/L) in the walnut tolerant group, compared to PbP median diameter of 5.0 mm (IQR 3.0 mm) and median sIgE of 4.0 kUA/L (IQR 11.1 kUA/L) in the walnut allergic group. Sensitivity, specificity, positive and negative predictive values and likelihood ratio are shown in Table 1. ROC curves for PbP (area under curve 0.665, *p* = 0.0092) and sIgE (area under curve 0.688, *p* = 0.0075) are shown in Figure 1.

**TABLE 1:** Sensitivity, specificity, positive and negative predictive values and positive likelihood ratio. The median values of PbP and sIgE to walnut were studied in the walnut tolerant group and in the walnut allergic group. Hence, we identified the cut‐off values for PbP and sIgE with the higher sensitivity, specificity, positive predictive value (PPV) and negative predictive value (NPV) as well as sensitivity, specificity, PPV and NPV to other potential cut‐off values.

	Sensitivity (95% CI)	Specificity (95% CI)	PPV (95% CI)	NPV (95% CI)	Positive likelihood ratio (95% CI)
sIgE: cut‐off > 0.10 kUa/L	1.00 (1.00–1.00)	0.11 (0.02–0.20)	0.52 (0.41–0.63)	1.00 (1.00–1.00)	1.13 (1.12–1.13)
sIgE: cut‐off > 0.60 kUa/L	0.91 (0.82–0.99)	0.29 (0.16–0.42)	0.55 (0.43–0.67)	0.76 (0.56–0.97)	1.28 (1.26–1.29)
sIgE: cut‐off > 2.50 kUa/L	0.67 (0.53–0.81)	0.60 (0.46–0.74)	0.62 (0.48–0.76)	0.66 (0.51–0.80)	1.69 (1.65–1.72)
sIgE: cut‐off > 18.50 kUa/L	0.21 (0.09–0.33)	0.93 (0.86–1.00)	0.75 (0.51–1.00)	0.55 (0.44–0.66)	3.14 (2.95–3.34)
PbP: cut‐off > 3 mm	0.86 (0.76–0.96)	0.42 (0.28–0.57)	0.59 (0.47–0.71)	0.76 (0.59–0.93)	1.49 (1.47–1.51)
PbP: cut‐off > 4 mm	0.74 (0.61–0.87)	0.51 (0.37–0.66)	0.59 (0.46–0.72)	0.68 (0.52–0.83)	1.52 (1.50–1.55)
PbP: cut‐off > 5 mm	0.47 (0.32–0.61)	0.71 (0.58–0.84)	0.61 (0.44–0.77)	0.58 (0.45–0.71)	1.61 (1.57–1.66)
PbP: cut‐off > 7 mm	0.19 (0.07–0.30)	0.91 (0.83–0.99)	0.67 (0.40–0.93)	0.54 (0.43–0.65)	2.09 (1.98–2.21)

Abbreviations: CI, confidence interval; NPV, negative predictive value; PbP, prick‐by‐prick; PPV, positive predictive value; sIgE, specific IgE.

**FIGURE 1:**
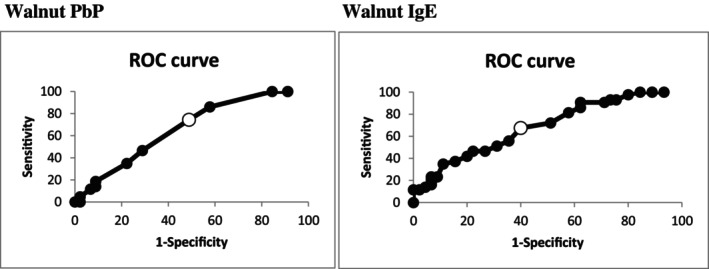
ROC curves walnut PbP and walnut sIgE. Receiver operating characteristic (ROC) curve analyses were assessed for sIgE and PbP. Correlation between PbP and sIgE to walnut was performed through linear regression and Spearman correlation index. Besides, we took into consideration the role of age, sex, PbP or sIgE values in association with positive OFCs. Finally, we studied the relevance of clinical history in association with positive OFCs to walnut when it was reported as the trigger food. A multiple logistic regression analysis was carried out with the possibility of reaction as an outcome variable and the demographic and clinical variables as covariates. Results were quantified through adjusted odds ratio (aOR) and a 95% Wald confidence interval (CI). Analyses utilised SAS (version 9.4; SAS Institute, Cary). The Spearman correlation coefficient between PbP and sIgE was 0.39, pointing out a positive correlation (0.23 for the walnut allergic group, 0.41 for the walnut tolerant group). The multiple logistic regression analysis showed that PbP and sIgE are independent predictors of risk for reaction to walnut OFC. We observed that in children of the same age, sex and history of reaction, odds of reaction increase with each mm increase in walnut PbP (aOR: 1.23; 95% Wald CI 1.01–1.50, *p* < 0.05), and with each unit increase in sIgE (aOR: 1.05; 95% Wald CI 1.00–1.09, *p* = 0.06). PbP, prick‐by‐prick; ROC, receiver operating characteristic; sIgE, specific IgE.

The diagnostic test accuracy of PbP for walnut allergy has not previously been studied, and to our knowledge, only a few studies evaluated sIgE [4]. In our study, walnut allergy was formally excluded or confirmed by OFC with the trigger allergen.

Limitations include the single‐centre, retrospective design, lack of blinding of OFC interpretation with respect to the result of the PbP and sIgE and the risk of a selection bias. Finally, we did not explore the diagnostic test accuracy of Jug r 1 or Jug r 3 as component allergens and skin prick testing using commercial walnut extract. We recorded a low rate of anaphylaxis at OFC, with a moderate rate of intramuscular adrenaline use.

In conclusion, we have developed diagnostic cut‐offs for walnut allergy, which may support the identification of patients for OFC in the diagnostic assessment of walnut allergy. PbP seems to have a similar diagnostic performance compared with sIgE, slightly better for sIgE for diagnosing walnut allergy in children. However, the diagnostic cut‐offs identified in our study need validation in other populations.

## Author Contributions

C.F. collected the data. M.G., M.T., G.L., L.S., S.B., L.T., B.P., C.V., and F.M. performed the investigations. C.F. and R.P. analysed the data. C.F., R.P., and M.G. drafted the initial manuscript. M.G., C.F., M.T., G.L., L.S., S.B., L.T., B.P., C.V., R.B., R.P., and F.M. interpreted the data and reviewed the manuscript. All authors approved the final manuscript as submitted and agreed to be accountable for all aspects of the work.

## Ethics Statement

Written informed consent was obtained from the children's parents for all procedures performed. The code of the event report issued by Meyer Children's University Hospital is IR904‐23‐65903.

## Conflicts of Interest

M.G. reports personal fees from Sanofi (educational events). S.B. reports personal fees from Sanofi and Nutricia (educational events).

## Data Availability

Aggregate analyses are available on reasonable request to the corresponding author.
